# (*E*,*E*)-*N*,*N*′-Bis­(4-methoxy­benzyl­idene)cyclo­hexane-1,2-diamine

**DOI:** 10.1107/S1600536808023751

**Published:** 2008-07-31

**Authors:** Guo-Xi Wang

**Affiliations:** a Department of Chemical Engineering, Anyang Institute of Technology, Anyang, 455000, People’s Republic of China

## Abstract

In the title compound, C_22_H_26_N_2_O_2_, the meth­oxy and the benzyl­idene groups are essentially coplanar, and the cyclo­hexane ring has a chair conformation. The two halves of the mol­ecule are related by a twofold rotation. The crystal structure is stabilized only by van der Waals inter­actions.

## Related literature

For the chemistry of Schiff base derivatives, see: Negm & Zaki (2008 ▶); Feng *et al.* (2008 ▶); Lee & Do (2007 ▶).
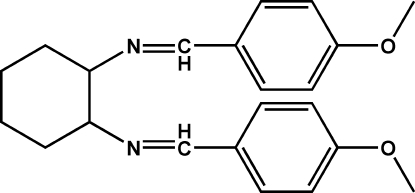


## Experimental

### 

#### Crystal data

C_22_H_26_N_2_O_2_
*M* *_r_* = 350.45
Orthorhombic, 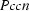

*a* = 19.674 (3) Å
*b* = 5.4097 (9) Å
*c* = 18.662 (3) Å
*V* = 1986.2 (6) Å^3^
*Z* = 4
Mo *K*α radiation
μ = 0.07 mm^−1^
*T* = 298 (2) K
0.35 × 0.30 × 0.20 mm

#### Data collection

Rigaku Mercury2 diffractometer
Absorption correction: multi-scan (*CrystalClear*; Rigaku, 2005 ▶) *T*_min_ = 0.978, *T*_max_ = 0.985
18603 measured reflections
2262 independent reflections
1588 reflections with *I* > 2σ(*I*)
*R*_int_ = 0.060

#### Refinement

*R*[*F* ^2^ > 2σ(*F* ^2^)] = 0.066
*wR*(*F* ^2^) = 0.154
*S* = 1.12
2262 reflections
118 parameters
H-atom parameters constrained
Δρ_max_ = 0.13 e Å^−3^
Δρ_min_ = −0.14 e Å^−3^

### 

Data collection: *CrystalClear* (Rigaku, 2005 ▶); cell refinement: *CrystalClear*; data reduction: *CrystalClear*; program(s) used to solve structure: *SHELXS97* (Sheldrick, 2008 ▶); program(s) used to refine structure: *SHELXL97* (Sheldrick, 2008 ▶); molecular graphics: *SHELXTL* (Sheldrick, 2008 ▶); software used to prepare material for publication: *SHELXTL* .

## Supplementary Material

Crystal structure: contains datablocks I, New_Global_Publ_Block. DOI: 10.1107/S1600536808023751/wk2089sup1.cif

Structure factors: contains datablocks I. DOI: 10.1107/S1600536808023751/wk2089Isup2.hkl

Additional supplementary materials:  crystallographic information; 3D view; checkCIF report

## Figures and Tables

**Fig. 1.**
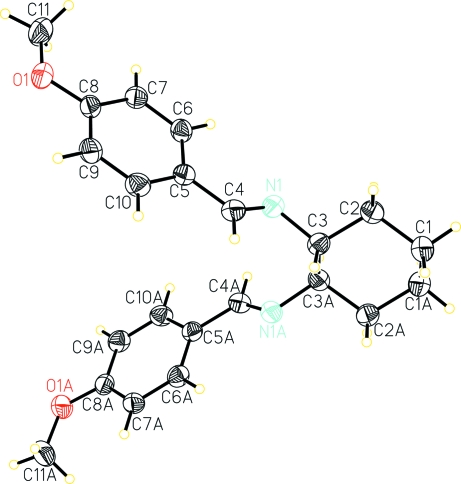
A view of the title compound with the atom numbering scheme, symmetry related atoms, (-x+1/2, -y+1/2, z) denoted by A. Displacement ellipsoids were drawn at the 30% probability level.

## supplementary crystallographic information

### Comment

In the past five years, we have focused on the chemistry of schiff-base derivatives because of their biological behaviors and their multiple coordination modes as ligands to metal ions and for the construction of novel metal-organic frameworks (Negm & Zaki,  2008; Feng *et al.* 2008; Lee & Do, 2007). We report here the crystal structure of the title compound, (*N*^1^*E*,*N*^2^*E*)-*N*^1^,*N*^2^-bis(4-methoxybenzylidene)cyclohexane-1,2-diamine.

In the title compound (Fig.1), there is a rotation axis bisecting the molecule through the cyclohexane ring. The methoxy  and the benzylidene groups are essentially coplanar, and the cyclohexane-1,2-diamine group is in the chair form. The C4=N1 bond length of 1.249 (2) Å is consistent with the value for a double bond. The crystal structure is stabilized only by van der Waals interactions.

### Experimental

*rac*-Diaminocyclohexane (1.20 g, 10.5 mmol) and *p*-anisaldehyde (1.36 g, 10.0 mmol) were dissolved in ethanol (20 mL) under magnetic stirring. The mixture was heated to reflux for 12 h. After cooled to room temperature, the resulting content was put to a refrigerator to stand over night. Then, the precipitate was filtered off and recrystallized from ethanol affording colorless block crystals of this compound suitable for X-ray analysis were obtained.

### Refinement

All H atoms attached to C atoms were fixed geometrically and treated as riding with C–H = 0.93 Å(aromatic), C–H = 0.98 Å(methine), 0.97 Å(methylene), C–H = 0.96 Å(methyl), with *U*_iso_(H) =1.2U_eq_(C except methyl C) and *U*_iso_(H) =1.5U_eq_(methyl C).

### Crystal data

**Table:**

C_22_H_26_N_2_O_2_	*F*_000_ = 752
*M**_r_* = 350.45	*D*_x_ = 1.172 Mg m^−^^3^
Orthorhombic, *P**c**c**n*	Mo *K*α radiation λ = 0.71073 Å
Hall symbol: -P 2ab 2ac	Cell parameters from 3131 reflections
*a* = 19.674 (3) Å	θ = 3.0–27.4º
*b* = 5.4097 (9) Å	µ = 0.08 mm^−^^1^
*c* = 18.662 (3) Å	*T* = 298 (2) K
*V* = 1986.2 (6) Å^3^	Block, colorless
*Z* = 4	0.35 × 0.30 × 0.20 mm

### Data collection

**Table:**

Rigaku Mercury2 diffractometer	2262 independent reflections
Radiation source: fine-focus sealed tube	1588 reflections with *I* > 2σ(*I*)
Monochromator: graphite	*R*_int_ = 0.060
Detector resolution: 13.6612 pixels mm^-1^	θ_max_ = 27.5º
*T* = 298(2) K	θ_min_ = 3.0º
ω scans	*h* = −25→25
Absorption correction: multi-scan(CrystalClear; Rigaku, 2005)	*k* = −7→7
*T*_min_ = 0.978, *T*_max_ = 0.985	*l* = −24→24
18603 measured reflections	

### Refinement

**Table:**

Refinement on *F*^2^	Secondary atom site location: difference Fourier map
Least-squares matrix: full	Hydrogen site location: inferred from neighbouring sites
*R*[*F*^2^ > 2σ(*F*^2^)] = 0.066	H-atom parameters constrained
*wR*(*F*^2^) = 0.154	*w* = 1/[σ^2^(*F*_o_^2^) + (0.0572*P*)^2^ + 0.4317*P*] where *P* = (*F*_o_^2^ + 2*F*_c_^2^)/3
*S* = 1.12	(Δ/σ)_max_ < 0.001
2262 reflections	Δρ_max_ = 0.13 e Å^−^^3^
118 parameters	Δρ_min_ = −0.14 e Å^−^^3^
Primary atom site location: structure-invariant direct methods	Extinction correction: none

### Special details

**Table:**

Geometry. All e.s.d.'s (except the e.s.d. in the dihedral angle between two l.s. planes) are estimated using the full covariance matrix. The cell e.s.d.'s are taken into account individually in the estimation of e.s.d.'s in distances, angles and torsion angles; correlations between e.s.d.'s in cell parameters are only used when they are defined by crystal symmetry. An approximate (isotropic) treatment of cell e.s.d.'s is used for estimating e.s.d.'s involving l.s. planes.
Refinement. Refinement of *F*^2^ against ALL reflections. The weighted *R*-factor *wR* and goodness of fit *S* are based on *F*^2^, conventional *R*-factors *R* are based on *F*, with *F* set to zero for negative *F*^2^. The threshold expression of *F*^2^ > σ(*F*^2^) is used only for calculating *R*-factors(gt) *etc*. and is not relevant to the choice of reflections for refinement. *R*-factors based on *F*^2^ are statistically about twice as large as those based on *F*, and *R*- factors based on ALL data will be even larger.

### Fractional atomic coordinates and isotropic or equivalent isotropic displacement parameters (Å^2^)

**Table:**

	*x*	*y*	*z*	*U*_iso_*/*U*_eq_	
O1	0.47992 (8)	0.4625 (3)	0.41000 (8)	0.0713 (5)	
N1	0.32205 (8)	0.3062 (3)	0.11039 (9)	0.0558 (5)	
C5	0.36446 (9)	0.4561 (4)	0.22382 (10)	0.0493 (5)	
C2	0.32123 (10)	0.3262 (5)	−0.02019 (11)	0.0629 (6)	
H2A	0.3454	0.1699	−0.0204	0.075*	
H2B	0.3547	0.4577	−0.0203	0.075*	
C7	0.45135 (10)	0.2643 (4)	0.29659 (11)	0.0535 (5)	
H7	0.4827	0.1380	0.3034	0.064*	
C4	0.32303 (10)	0.4677 (4)	0.15823 (10)	0.0553 (5)	
H4	0.2955	0.6057	0.1519	0.066*	
C8	0.44380 (10)	0.4485 (4)	0.34762 (10)	0.0513 (5)	
C6	0.41180 (10)	0.2704 (4)	0.23553 (10)	0.0539 (5)	
H6	0.4170	0.1467	0.2014	0.065*	
C3	0.27888 (9)	0.3439 (4)	0.04791 (10)	0.0532 (5)	
H3	0.2592	0.5101	0.0506	0.064*	
C10	0.35804 (10)	0.6392 (4)	0.27558 (11)	0.0591 (5)	
H10	0.3270	0.7666	0.2688	0.071*	
C1	0.27826 (11)	0.3452 (4)	−0.08769 (11)	0.0636 (6)	
H1B	0.2587	0.5095	−0.0908	0.076*	
H1A	0.3069	0.3204	−0.1294	0.076*	
C11	0.52945 (14)	0.2748 (6)	0.42442 (13)	0.0871 (8)	
H11A	0.5509	0.3076	0.4696	0.131*	
H11B	0.5631	0.2746	0.3872	0.131*	
H11C	0.5075	0.1164	0.4262	0.131*	
C9	0.39688 (10)	0.6346 (4)	0.33659 (11)	0.0595 (6)	
H9	0.3916	0.7580	0.3708	0.071*	

### Atomic displacement parameters (Å^2^)

**Table:**

	*U*^11^	*U*^22^	*U*^33^	*U*^12^	*U*^13^	*U*^23^
O1	0.0718 (9)	0.0912 (12)	0.0509 (9)	0.0097 (9)	−0.0096 (7)	−0.0121 (8)
N1	0.0534 (9)	0.0608 (11)	0.0532 (10)	0.0022 (8)	−0.0042 (8)	−0.0036 (8)
C5	0.0448 (9)	0.0533 (11)	0.0497 (11)	−0.0015 (9)	0.0020 (8)	−0.0005 (9)
C2	0.0514 (11)	0.0790 (16)	0.0583 (13)	−0.0074 (10)	0.0038 (10)	0.0038 (11)
C7	0.0538 (11)	0.0521 (12)	0.0544 (11)	0.0079 (9)	0.0001 (10)	−0.0009 (9)
C4	0.0490 (11)	0.0572 (12)	0.0597 (13)	0.0051 (9)	−0.0020 (9)	0.0010 (10)
C8	0.0510 (10)	0.0593 (12)	0.0436 (10)	−0.0043 (9)	0.0035 (9)	−0.0025 (9)
C6	0.0574 (11)	0.0524 (12)	0.0518 (11)	0.0006 (9)	0.0032 (10)	−0.0101 (9)
C3	0.0528 (11)	0.0543 (12)	0.0524 (11)	0.0028 (9)	−0.0044 (9)	0.0016 (9)
C10	0.0572 (12)	0.0541 (12)	0.0661 (13)	0.0088 (10)	0.0001 (10)	−0.0060 (10)
C1	0.0680 (13)	0.0694 (14)	0.0535 (12)	−0.0003 (11)	0.0036 (10)	0.0037 (10)
C11	0.0886 (17)	0.110 (2)	0.0625 (15)	0.0236 (16)	−0.0193 (13)	0.0001 (14)
C9	0.0628 (12)	0.0580 (13)	0.0578 (13)	0.0034 (10)	0.0017 (10)	−0.0148 (10)

### Geometric parameters (Å, °)

**Table:**

O1—C8	1.366 (2)	C4—H4	0.9300
O1—C11	1.433 (3)	C8—C9	1.381 (3)
N1—C4	1.249 (2)	C6—H6	0.9300
N1—C3	1.457 (2)	C3—C3^i^	1.525 (4)
C5—C10	1.389 (3)	C3—H3	0.9800
C5—C6	1.387 (3)	C10—C9	1.371 (3)
C5—C4	1.472 (3)	C10—H10	0.9300
C2—C3	1.523 (3)	C1—C1^i^	1.516 (4)
C2—C1	1.521 (3)	C1—H1B	0.9700
C2—H2A	0.9700	C1—H1A	0.9700
C2—H2B	0.9700	C11—H11A	0.9600
C7—C6	1.380 (3)	C11—H11B	0.9600
C7—C8	1.386 (3)	C11—H11C	0.9600
C7—H7	0.9300	C9—H9	0.9300
			
C8—O1—C11	118.35 (17)	N1—C3—C2	109.88 (15)
C4—N1—C3	118.88 (17)	C3^i^—C3—C2	111.46 (14)
C10—C5—C6	117.90 (18)	N1—C3—H3	108.5
C10—C5—C4	119.83 (18)	C3^i^—C3—H3	108.5
C6—C5—C4	122.25 (18)	C2—C3—H3	108.5
C3—C2—C1	112.52 (16)	C9—C10—C5	120.92 (19)
C3—C2—H2A	109.1	C9—C10—H10	119.5
C1—C2—H2A	109.1	C5—C10—H10	119.5
C3—C2—H2B	109.1	C1^i^—C1—C2	111.22 (15)
C1—C2—H2B	109.1	C1^i^—C1—H1B	109.4
H2A—C2—H2B	107.8	C2—C1—H1B	109.4
C6—C7—C8	119.32 (19)	C1^i^—C1—H1A	109.4
C6—C7—H7	120.3	C2—C1—H1A	109.4
C8—C7—H7	120.3	H1B—C1—H1A	108.0
N1—C4—C5	124.96 (19)	O1—C11—H11A	109.5
N1—C4—H4	117.5	O1—C11—H11B	109.5
C5—C4—H4	117.5	H11A—C11—H11B	109.5
O1—C8—C9	115.75 (17)	O1—C11—H11C	109.5
O1—C8—C7	124.70 (18)	H11A—C11—H11C	109.5
C9—C8—C7	119.55 (18)	H11B—C11—H11C	109.5
C7—C6—C5	121.71 (18)	C8—C9—C10	120.61 (19)
C7—C6—H6	119.1	C8—C9—H9	119.7
C5—C6—H6	119.1	C10—C9—H9	119.7
N1—C3—C3^i^	109.94 (14)		
			
C3—N1—C4—C5	−179.41 (17)	C4—N1—C3—C3^i^	−112.3 (2)
C10—C5—C4—N1	−175.8 (2)	C4—N1—C3—C2	124.6 (2)
C6—C5—C4—N1	6.0 (3)	C1—C2—C3—N1	175.80 (18)
C11—O1—C8—C9	−179.9 (2)	C1—C2—C3—C3^i^	53.7 (3)
C11—O1—C8—C7	0.1 (3)	C6—C5—C10—C9	−0.6 (3)
C6—C7—C8—O1	−179.97 (18)	C4—C5—C10—C9	−178.92 (19)
C6—C7—C8—C9	0.0 (3)	C3—C2—C1—C1^i^	−54.7 (3)
C8—C7—C6—C5	−0.1 (3)	O1—C8—C9—C10	179.73 (18)
C10—C5—C6—C7	0.4 (3)	C7—C8—C9—C10	−0.3 (3)
C4—C5—C6—C7	178.64 (18)	C5—C10—C9—C8	0.6 (3)

Symmetry codes: (i) −*x*+1/2, −*y*+1/2, *z*.
